# The Coating of the Tongue

**Published:** 1871-01

**Authors:** 


*The Coating of the Tongue.*—Dr. J. M. DaCosta, in *Medical Diagnosis*, notes the following variations of the coating of the tongue: "In health the tongue has hardly a discernible lining; disease quickly gives it one. In inflammation of the respiratory textures, at the commencement of fevers, in disorders of large portions of the abdominal mucous tract, the epithelium accumulates, and the tongue has a loaded, whitish appearance. The coat is apt to be yellowish in disturbances of the liver, and of a brown or very dark hue when the blood is contaminated. But we must be very sure, in drawing our inferences, that the abnormal aspect be not due to the food partaken of, or to medicine. Its color is also modified by the character of the occupation. Thus, as Chambers asserts, there is a curious smooth, orange tinted coating on the tongue of tea tasters. A local cause sometimes gives rise to a thick opaque coat. For instance, decayed teeth may produce a yellow sheathing on one side. Affections of the fauces also occasion a deep yellow hue. Again, some persons, even in health, wake up every morning with their tongues covered at the back with a heavy coating, which wears off during the day."

